# Visualizing Person-centered Long-term Care: An Exploratory Scoping Review and Evidence Mapping

**DOI:** 10.1093/geroni/igab046.3549

**Published:** 2021-12-17

**Authors:** Mary Lou Ciolfi, Rocky Coastlines, Catherine Taylor, Kayla Thompson, Jennifer Crittenden, Lenard Kaye, Angela Hunt, Paul Nebenzahl

**Affiliations:** 1 University of Maine, Center on Aging, Bath, Maine, United States; 2 University of Maine Center on Aging, Bangor, ME 04401-4324, Maine, United States; 3 University of Maine Center on Aging, Bangor, Maine, United States; 4 University of Maine, Bangor, Maine, United States; 5 University of Maine, University of Maine, Maine, United States; 6 The Cedars, Portland, Maine, United States; 7 The Mayer-Rothschild Foundation, Chicago, Illinois, United States

## Abstract

Despite decades of professional, academic, and policy interest in person-centered long-term care (LTC), the field continues to be challenged by the absence of a comprehensive depiction of the concept and a lack of consistency reflected across studies and measures. In response, a participatory action, research-focused, partnership between an institution of higher education and an LTC community (the University of Maine Center on Aging and The Cedars), with funding from The Mayer-Rothschild Foundation, is identifying and mapping the landscape of person-centered LTC in nursing homes and assisted living communities. A collaborative, ongoing, exploratory scoping literature review and evidence mapping has compiled a database of 663 academic and 115 grey literature articles through 65 systematic searches reviewing over 4,296 articles. An iterative process from both the resident and organizational perspectives revealed nine core domains (e.g. dining, resident care, environment, quality improvement, identity and personhood, etc.) and two substantive research gaps (the intersection of person-centered long term care with diversity, equity, and inclusion issues, and pandemic considerations). For mapping purposes, domain content was analyzed categorically based on concept, information revealed about resident, family, and staff experience, and operations applicability. The identified person-centered LTC domains, categorical analysis, identified gaps, and visual representation via mapping will contribute to generating research ideas, supporting the development of effective operationalization for LTC settings, and contributing to an understanding of the theoretical scope and concrete elements of a person-centered care model that aims to improve the wellbeing and quality of life of older adults in long-term residential settings.

